# Positive feedback loop of miR-320 and CD36 regulates the hyperglycemic memory-induced diabetic diastolic cardiac dysfunction

**DOI:** 10.1016/j.omtn.2023.03.019

**Published:** 2023-04-15

**Authors:** Esko Kankuri

**Affiliations:** 1 Department of Pharmacology, Faculty of Medicine, University of Helsinki, Helsinki, Finland

The term "metabolic memory" was coined by the investigators of the Diabetes Control and Complications Trial (DCCT) and the Epidemiology of Diabetes Interventions and Complications (EDIC) study.^1^ During the DCCT, patients on actively controlled and intensively modified insulin therapy achieved median glycated hemoglobin levels of 7% compared with 9% in patients on more conventional insulin therapy. The DCCT was terminated 1 year ahead of its completion due to clear outcomes, which included reduction of early-stage microvascular complications by 35%–76%, in favor of intensive therapy. The EDIC observational study was then initiated as the DCCT follow-up, and all participants were offered training in the intensive treatment principles. During the EDIC follow-up study, the differences in glycated hemoglobin levels between the study groups of the DCCT disappeared. However, durable effects on microvascular complication outcomes were observed between the original treatment groups of the DCCT. This metabolic memory was assigned to long-lasting glycation of mitochondrial proteins leading to increased production of reactive oxygen species (ROS).^2^ Long-term follow-up studies on hyperglycemic memory have provided further insight into this observation, also termed the legacy effect.^3^ However, although hyperglycemic memory links good glycemic control in early diabetes with less microvascular complications, vascular changes and other diabetes complications that accumulate over time can mute the effect.^3^

The burden of diabetes is manifested in its several associated complications. With improved disease management and increased life years, complications such as micro- and macrovascular diseases, such as retinopathy, neuropathy, coronary heart disease, and diabetic kidney disease, are accompanied by emerging links and increased susceptibility of diabetics to, for example, cancer and cognitive disabilities.^4^ On a global scale, it has been difficult to grasp the full extent of the diabetes problem. This is manifested, for example, by the immense variability of the prevalence estimates for type 1 diabetes (T1D). All-age estimates of DM1 prevalence range from 8.4 million to a staggering 22 million.^5^^,^^6^ Moreover, there are indications that the T1D prevalence may be expected to double by 2040.^5^ Including type 2 diabetes (T2D), the overall global prevalence of diabetes is estimated between ∼460 and ∼538 million individuals.^6^^,^^7^ In particular, the incidence of T2D is increasing in low- and middle-income countries. These numbers emphasize the need for improving our understanding of diabetes, its early diagnosis, and effective management to limit complications and disease burden.

Hyperglycemia, insulin resistance, and obesity all contribute to the development of cardiac structural and functional alterations frequently observed in diabetic patients. Cardiac dysfunction affects 14.5% of individuals with T1D, and its prevalence is more than double, up to 35%, in T2D.^8^ Poor glycemic control, evaluated by every 1% increase in glycated hemoglobin, increases the risk of heart failure in T1D by 30%. In T2D, further complicated with other metabolic disturbances, every percent increase in glycated hemoglobin adds 8% to the heart failure risk.^8^ Diabetic heart failure frequently first manifests as isolated diastolic dysfunction. In T2D, heart failure is more typically characterized by poor cardiac compliance, increased systemic and pulmonary congestion, and preserved systolic function, while in T1D it is more frequently accompanied by symptoms of systolic dysfunction.^8^ The development of diabetic cardiac dysfunction, at the molecular level, is linked to a wide variety of mechanisms including increased expression of the fatty acid translocase, CD36, and its localization to the cell surface and sarcolemma membranes. Increased CD36 contributes to cardiomyocyte damage by driving fatty acid intake, cellular lipid accumulation, and lipotoxicity.^8^ As our understanding of the mechanisms of diabetes complications and the intertwined relationship between glycemic control and fatty acid metabolism increases, we can expect improved therapeutic options to emerge with which to reinstate control over disrupted glucose balance, avoid diabetes-associated complications, and reduce morbidity.

To this end, Zhan et al.^9^ addressed the molecular mechanisms of cardiac hyperglycemic memory in detail. Building on their previous results^10^ on cardiac miRNA profiling in a genetic mouse model of diabetes-induced cardiac dysfunction and comparing these data with their previous analysis of circulating miRNAs in patients with heart failure, the authors focused on miR-320. In their previous work, the authors had demonstrated that miR-320 expression was increased in the failing hearts of diabetic mice.^10^ Administration of a tough decoy (TuD) miR-320 inhibitor using cardiac-selective adeno-associated virus serotype 9 (AAV9) counteracted development of cardiac functional defects and reduced myocardial apoptosis.^10^ Followed by their discovery of high-glucose and palmitate-induced miR-320 nuclear localization and co-localization with argonaute-2 (AGO2), an integral part of the RNA-induced silencing complex, Li et al.^10^ found miR-320 to increase the expression of VLDLR and CD36 in cardiomyocytes. A similar expression pattern was found in diabetic mice hearts, and miR-320 inhibition was able to decrease the increased expressions of CD36 and VLDLR. Most interestingly, cardiac overexpression of CD36 led to an increase in miR-320 expression *in vivo*. After demonstrating that miR-320 stimulates cardiomyocyte uptake of free fatty acids, the authors demonstrated that miR-320 directly binds to the promoter of the *Cd36* gene and activates its transcription. The authors concluded that, by activating *Cd36* transcription, miR-320 increases cardiomyocyte fatty acid intake and thus contributes to cardiomyocyte apoptosis through increased lipotoxicity, production of ROS, and decreased mitochondrial ATP production. Interestingly, also the FA-binding protein 3 (FABP3), a marker of cardiac injury associated with adverse cardiac outcomes such as congestive heart failure and recurrent myocardial infarction,^11^ was found to be a target for miR-320 transcriptional activation. The contribution of the miR-320-FABP3 axis to cardiac damage would deserve further investigations.

**Figure 1:**
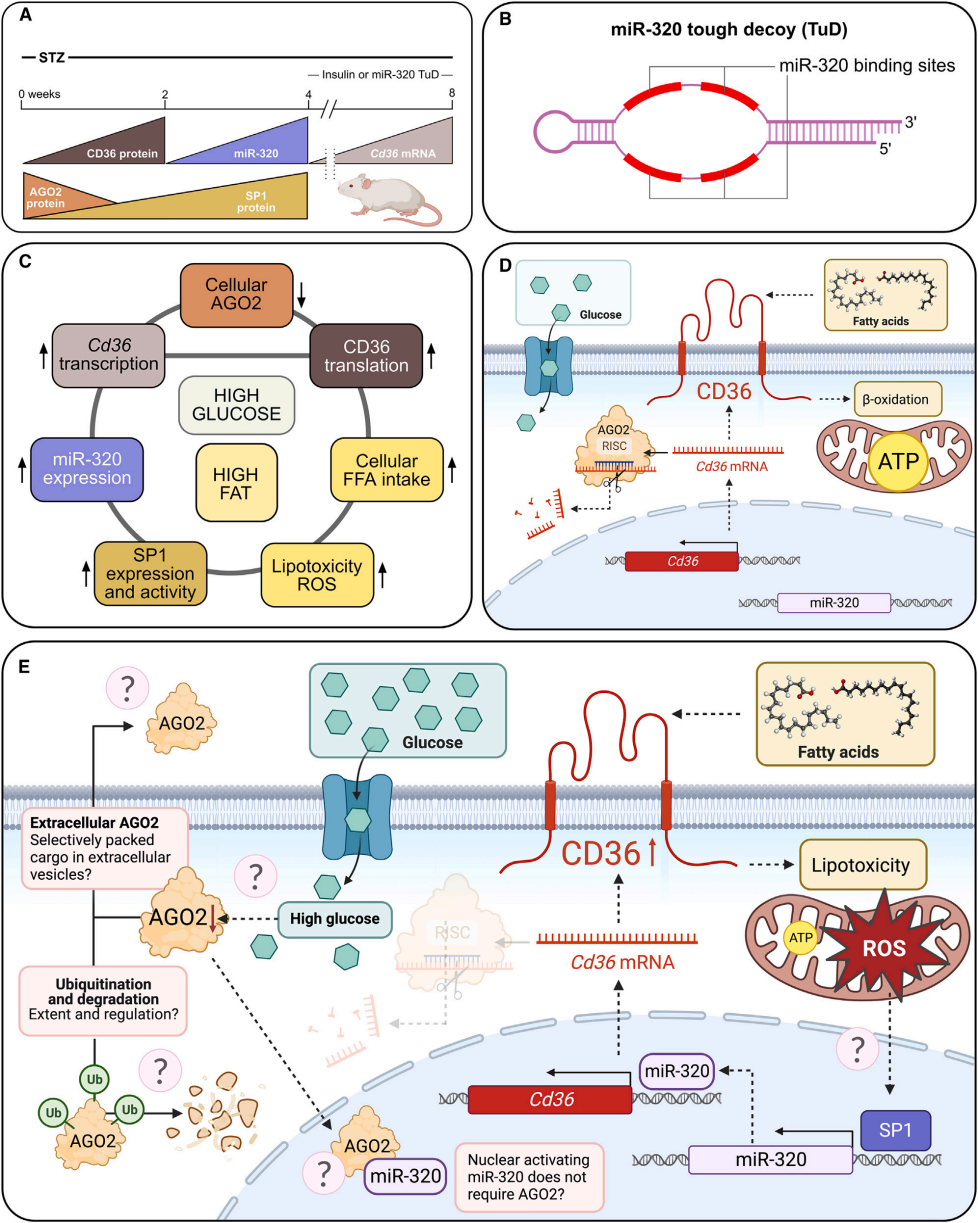
Summary of results and outstanding questions regarding the work by Zhan et al. (A) Time course of the molecular findings and timing of therapy from Zhan et al.^9^ in the streptozotocin (STZ)-induced diabetes model. (B) Schematic visualization of miR-320 tough decoy (TuD) and its miR-320 binding sites. (C) Graphical overview into the results by Zhan et al. (D) Translation of CD36 is controlled post-transcriptionally by AGO2/RNA-induced silencing complex (RISC), which targets *Cd36* mRNA for degradation. CD36 translocates free fatty acids for subsequent beta-oxidation and production of ATP in the mitochondria. (E) High glucose induces loss of AGO2 by both extracellular release and ubiquitination followed by proteolysis. Increased CD36 protein is produced after Ago2/RISC no longer attacks its mRNA. Ample amounts of CD36 readily harvest free fatty acids to the cell causing accumulation of lipids, lipotoxicity, apoptosis, and mitochondrial production of reactive oxygen species (ROS). ROS-induced signaling increases expression and activity of the SP1 transcription factor leading to increased transcription of miR-320. Nuclear miR-320 subsequently binds to the *Cd36* gene promoter and activates *Cd36* transcription, thus further enhancing CD36 expression and intracellular harvesting of free fatty acids. This figure was created using BioRender.com.

Now, Zhan et al.^9^ first set out to evaluate the ability of glycemic control to abate diabetes-induced diastolic dysfunction. In a model of streptozotocin (STZ)-induced diabetes, the authors, however, found that normalizing the STZ-induced increase in blood glucose by insulin, administered for 4 weeks either 1 or 2 months after STZ induction of diabetes, could neither alleviate diastolic cardiac dysfunction nor reduce cardiomyocyte lipid accumulation. In contrast, when administered instead of insulin, the miR-320 TuD inhibitor protected the mice against STZ-induced diabetic cardiac dysfunction and lipid accumulation. The authors also demonstrated cardioprotective effects of the miR-320 TuD inhibitor in the leptin receptor-deficient (db/db) mouse model of T2D. In cardiomyocytes, the first responder to high glucose was a decrease in AGO2, the protein levels of which demonstrated dramatic reductions already at 3 h in high-glucose-treated cardiomyocytes and at 2 weeks after STZ (Figures 1A–1C). In the hearts of STZ-induced diabetic mice, decreased AGO2 was mirrored by increased protein levels of CD36, both preceding the increases in miR-320 expression. At already 2 weeks after STZ, increases in CD36 protein expression were observed, while increases in cardiac miR-320 were found after 1 month followed by the increases in CD36 mRNA levels after 2 months. In more detailed mechanistic experiments on cardiomyocytes, knocking down *Ago2* gene expression resulted in increased CD36 protein levels, suggesting that the reduction in cellular AGO2 by high glucose increased the amounts of CD36 protein. Induced overexpression of CD36, on the other hand, increased not only the levels of miR-320 but also those of the transcription factor SP1. When the authors then silenced SP1 expression in cells overexpressing CD36, they were able to abolish the CD36-induced increase in miR-320, thus linking SP1 in the pathway leading from increased CD36 expression to increased miR-320. Stressing the cardiomyocyte cultures with high glucose reduced binding of CD36 mRNA to AGO2, but did not affect either *Cd36* or *Ago2* mRNA levels. Attempting to find mechanisms for the glucose-induced loss of AGO2 protein specifically, the authors discovered that, after glucose stress, increased amounts of AGO2 protein were actually found in the cell culture medium and also that AGO2 ubiquitination was increased intracellularly (Figures 1C–1E). Interestingly, an exploratory analysis of diabetic patient and control plasma samples revealed a diabetes-associated increase in plasma AGO2 protein as well.

The data presented by Zhan et al.^9^ offer the first evidence into high-glucose-responsive loss of intracellular AGO2 leading to *Cd36* mRNA degradation and increased CD36 protein translation. The increased levels of CD36 are then followed by increased nuclear expression of miR-320, which in turn activates *Cd36* gene expression, increases fatty acid accumulation, lipotoxicity, and generation of ROS. As CD36-overexpression-induced SP1 levels were abated by the antioxidant N-acetylcysteine, the authors suggested that ROS-induced SP1 could then drive the increase in miR-320 gene expression. This enticing loop or vicious circle of interactions, as now revealed by Zhan et al.,^9^ drives biomedical diabetes research forward and opens the rationale for AGO2-, CD36-, miR-320-, or SP1-targeted therapeutic approaches. As miR-320 has also been shown to increase cardiomyocyte apoptosis after cardiac ischemia reperfusion injury,^12^ the miR-320 TuD inhibitor may even have wider therapeutic potential. However, care should be taken to evaluate the tumor suppressor role of miR-320^13^ and rule out any adverse effects in this, or other, regards for decreasing in an untargeted manner the levels of miR-320. Targeting this therapy to the heart, nevertheless, holds great promise. Zhan et al. achieved this by using AAV9, which demonstrates cardiac tropism. Another interesting therapeutic approach stimulated by the work of Zhan et al. relates to the inhibition of CD36, for example, by targeting and inhibiting it using monoclonal antibodies.^14^ CD36 is also a cancer driver that has been implicated in promoting metastasis through fatty acid intake. Since cancer therapeutics are already designed to target it, CD36 inhibition in diastolic dysfunction would widen the therapeutic indication for these drugs. The similarities between molecular networks disrupted in cancer and metabolic disease as well as in degenerative diseases, are further evidenced by the involvement of the SP1 transcription factor in hyperglycemic memory, as presented by Zhan et al.^9^ Anti-cancer approaches targeting SP1 are under investigations,^15^ and SP1 inhibition has also demonstrated therapeutic efficacy in models of renal fibrosis and neurodegeneration.^16^^,^^17^

The results presented by Zhan et al.^9^ open intriguing research questions, and the mechanisms behind the relatively rapid loss of intracellular AGO2 and its putative co-operative loss-or-gain role with miR-320 for transcriptional activation necessitate further investigations in detail. Moreover, the contributions of mitochondrial fatty acid shuttling and relative inflexibility of fatty acid utilization as the preferred fuel in cardiomyocytes after the early neonatal period^18^ to diabetic cardiomyopathy and cardiac lipotoxicity remain to be resolved. As our understanding of the molecular mechanisms of poor-glycemic-control-induced changes increases and deepens, dissecting the disease-linked interregulatory networks and molecular pathways can be expected to open unprecedented new therapeutic opportunities for abating diabetic cardiomyopathy.
